# Outcome of deliveries in healthy but obese women: obesity and delivery outcome

**DOI:** 10.1186/1756-0500-6-50

**Published:** 2013-02-06

**Authors:** Rebecka Kaplan-Sturk, Helena Åkerud, Helena Volgsten, Lena Hellström-Westas, Eva Wiberg-Itzel

**Affiliations:** 1 Department of Clinical Science and Education, Section of Obstetrics and Gynecology, Karolinska Institute, Soder Hospital, Stockholm, 118 83, Sweden; 2 Department of Women’s and Children’s Health, Uppsala University, Uppsala, Sweden

**Keywords:** Obesity, Fetal outcome, Delivery outcome

## Abstract

**Background:**

Obesity among fertile women is a global problem. 25% of pregnant Swedish women are overweight at admission to the antenatal clinic and 12% of them are considered as obese. Previous studies have shown an increased risk of delivery complications with an elevated maternal BMI. The aim of this study was to evaluate delivery outcomes in relation to maternal BMI on admission to the antenatal clinic.

A healthy group of 787 women with full-term pregnancies and spontaneous onset of labor were included in the study. Delivery outcome was assessed in relation to maternal BMI when attending the antenatal clinic.

**Results:**

The results indicated that in deliveries where the maternal BMI was >30 a high frequency of abnormal CTG trace during the last 30 minutes of labor was shown. A blood sample for evaluation of risk of fetal hypoxia was performed in only eight percent of these deliveries. A spontaneous vaginal delivery without intervention was noted in 85.7%, and 12% of neonates were delivered with an adverse fetal outcome compared to 2.8% in the group with a maternal BMI<30 (p<0.001).

**Conclusion:**

These results indicate an increased risk at delivery for healthy, but obese women in labor. Furthermore, the delivery management may not always be optimal in these deliveries.

## Background

Obesity in women of childbearing age is a growing global problem. In Sweden, information on maternal height and weight on registration for antenatal care has been recorded since 1992, and onwards Body Mass Index (BMI, calculated as weight in kilograms divided by height in meters squared (kg/m^2^)) has increased among both primiparas as well as multiparas. In 2008, almost 25% of all pregnant women in Sweden were shown to be overweight (BMI 25-29.9) on presentation at the antenatal clinic and almost 12% of them were considered as obese (BMI ≥ 30) [1].

It is well known that obesity is associated with increased maternal and fetal morbidity during pregnancy and labour [2-8]. Furthermore, obesity is also associated with increased risk of caesarean section [3,5-15]. A meta-analysis has estimated the risk of caesarean section to be doubled for obese women, and tripled for women with severe obesity (BMI ≥ 35) [16]. An extended labour progress is associated with an increased risk of severe maternal and fetal outcome and has previously been shown to be overrepresented among women with obesity [17]. In addition, neonates born to obese mothers have a greater need for admission to neonatal intensive care [5,9,18]. The aim of this study was to evaluate maternal and neonatal outcome at delivery related to maternal BMI on admission to the antenatal clinic.

## Methods

**Figure 1:**
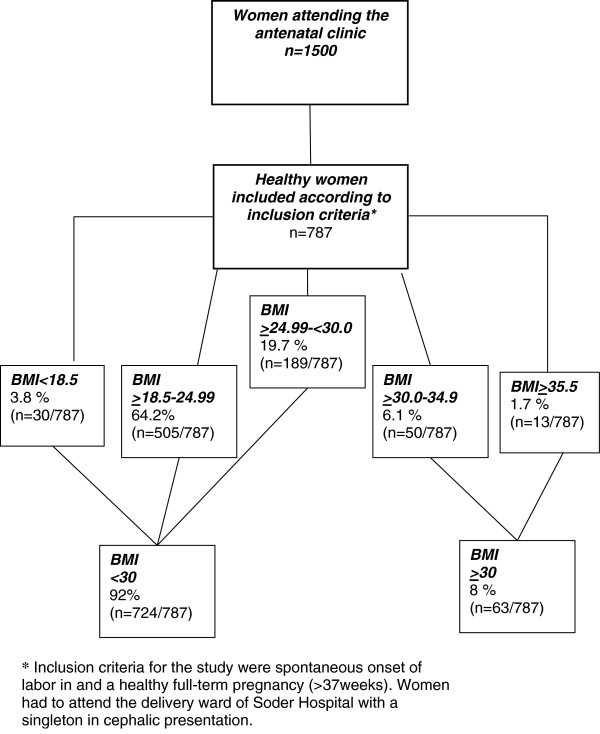
Flow chart over included deliveries.

This study was performed in Soder Hospital in Stockholm, Sweden, between November 2006 and May 2008. During the study period, approximately 1500 healthy women were eligible for inclusion in the study. Data on full maternal length and weight was recorded when attending the antenatal clinic at 10-12 weeks of gestation, and BMI was calculated according to the standard definition [1]. Finally, inclusion criteria for the study were spontaneous onset of labor and a healthy full-term pregnancy (≥37 weeks). Healthy women were in the study defined as women with no maternal complicated diseases as hypertension, preeclampsia, hepatoses, diabetes or other cronical diseases. Healthy feotuses were defined a foetuses with normal growth and no other complications. The women had to attend the delivery ward of Soder Hospital with a low-risk pregnancy and a singleton in cephalic presentation. 787 of the 1500 women fulfilled the inclusion criteria at onset of labour and were then actually included in the study. A written informed consent for participation in the study was obtained from all participants. The women included were then divided into two groups according to BMl when attending antenatal clinic; (BMI < 30 and BMI ≥ 30) Figure 1 shows a flow chart of the 787 women who participated in this analysis.

Labour dystocia was in this study diagnosed when labour progress passed the action line (AL) in the partogram (= primary dystocia) or if no progress of labour was made within 2 hours or more (= secondary dystocia).

All 787 deliveries included in the study underwent CTG monitoring during labour, 734 (93%) of these underwent continuous recording during the last 30 minutes before delivery. All CTG recordings were reviewed blind by two of the authors (EWI and HÅ). The review was made in accordance with the Federation of Gynaecology and Obstetrics (FIGO) guidelines for the use of fetal monitoring [19]. CTG recordings during the last 30 minutes before delivery were used in this calculation.

The umbilical cord was clamped immediately after delivery, before the newborn’s first cry. Arterial and venous blood samples were drawn from a double-clamped segment of the umbilical cord and pH, base deficit and lactate were analysed within a few minutes. All cord blood analyses were performed using the Bayer® automated Rapid Lab® 860 analyzer available in the labour wards. The base deficit was calculated from the blood compartment applying the algorithm used by Radiometer blood gas analyzers, recently reported to show a higher association with neonatal depression than base deficit calculated from the extra cellular fluid compartment [20]. As haemoglobin concentration in cord blood was not recorded, we used the general approximation of a haemoglobin concentration of 150 g/l.

The midwife, or the attending pediatrician, determined the Apgar score at 1, 5 and 10 minutes after delivery.

In 122 deliveries with a pathological CTG registration, the attending obstetrician decided to sample fetal scalp blood for lactate analysis [21], and the blood samples was analysed according to the hospital guidelines [21]. The attending obstetrician decided on the time and mode of delivery in foetuses with affected fetal blood samples (academia).

Symptoms closely associated with intrauterine asphyxia were in this study defined as adverse fetal outcome: a) pH < 7.10 in umbilical artery; and/or b) metabolic acidosis (i.e. pH < 7.05 and base deficit >12 in umbilical artery), and/or c) meconium aspiration, and/or d) transfer to neonatal intensive care (NICU).

Statistical analyses were performed using SPSS 17.0 (SPSS inc. Chicago, Illinois, USA), and the statistical Package Statistica for Windows, version 8.0 (Stat Soft, Tulsa, Oklahoma, USA). The study was approved by the regional ethics committee at Karolinska Institute, Stockholm (2006/718-31/3). The power calculation made for the study showed that at least 700 women had to be included to ensure clinical significance. The power calculation was performed in Sample Power 2.0. Background data of the population studied and fetal outcome were presented as frequencies (%), medians and range. Comparison between two continuous variables has been tested with the Mann–Whitney U- test. Proportions have been compared with the Chi- square test and in expected frequencies <5, Fisher’s exact test was used.

To estimate the association between different explanatory variables when the women attended the antenatal clinic and the odds of delivering a newborn with adverse fetal outcome we used logistic regression [22]. The explanatory variables measured were maternal age (> = 30, yes/no), parity (primiparous, yes/no), smoker (yes/no), BMI (> = 30 yes/no), gestational age (> = 41 weeks, yes/no, Table 1). The crude association of each explanatory variable with the odds of bad fetal outcome at delivery was also studied. The associations are presented as odds ratio (OR) with 95% confidence interval (CI).

**Table 1:** Different explanatory variables noted when attending antenatal clinic, and their association with an adverse fetal outcome at delivery

**Explanatory**	**Adverse fetal Outcome at delivery**	**Univariable OR (95% CI)**	**Multivariable OR (95% CI)**
Maternal Age ≥ 30			
No	14/342	Reference	
Yes	12/260	1.13 (0.5 -2.5)	
Primiparous			
No	7/231	Reference	
Yes	19/373	1.09 (0.96 – 1.3)	
Smoker			
No	24/564	Reference	
Yes	2/38	1.3 (0.3 – 5.5)	
BMI > =30			
No	20/554	Reference	Reference
Yes	6/50	3.6* (1.4-9.5)	3.6* (1.4-9.8)
BMI > = 35			
No	25/593	Reference	
Yes	1/11	0.4 (0.05-3.6)	
Gestational age			
<41 + 0 weeks	18/448	Reference	
≥41 + 0 weeks	8/156	1.3 (0.5 -3.0)	

Values are expressed as Odds Ratios (OR) with corresponding 95% confidence intervals (CI).

* P-values <0.05 were considered as statistically significant.

## Results

724 healthy women with a BMI <30 and 63 healthy women with a BMI ≥ 30, all with a normal pregnancy and a spontaneous onset of labour were included in the study. The median BMI measured when they attended the antenatal clinic was 22.8 (15.0-55.1) and the women’s age 31 years (18-46, Table 2). According to the BMI measured when attended the antenatal clinic, the deliveries were divided into two groups, maternal BMI <30 (n = 724) or ≥ 30 (n = 63, flowchart, Figure 1). Most women were primiparas (60%) and non-smokers (95.9%). The median gestational age at delivery in the group with a BMI < 30 was 282 days (259-300) and in the group with a BMI ≥ 30 281 (264 -296, p = 0.6, Table 2). Median birth weight in the group with a BMI < 30 was 3645 g (2320-5470) and in the group with BMI ≥ 30 3830 g (2720-4900, p = 0.05, Table 2).

**Table 2:** Background characteristics of the 787 mothers and infants included

	
**Maternal**	
Age (years)	31 (18-46)
Nullipara (%)	473 (60)
Smokers (%)	48 (6.1)
Height (cm)	167 (145-184)
Weight (kg)	63 (38-150)
BMI*	22.8 (15.0-55.1)
**Newborn**	
Birth weight (g)	3655 (2320-5470)
Gestational age (days)	282 (259-300)
Gender, boys (%)	379 (48)

Values are numbers (%) or medians (range).

*Body Mass Index, calculated as weight in kilograms divided by height in meters squared (Kg/m^2^).

No significant difference in length of delivery, i.e. labour dystocia (p = 0.3), the use of epidural anaesthetics (p = 0.2) or stimulation with oxytocin was identified between the two groups (p = 0.7). A significant difference in method of delivery was shown (p = 0.03). Spontaneous vaginal deliveries were more common in the group with a maternal BMI ≥ 30 compared to the group with a lower BMI (Table 3).

**Table 3:** Delivery characteristics in women with a BMI < 30 compared to women with a BMI > =30 (n = 787)

	**(BMI < 30) (n = 724)**	**(BMI > =30) (n = 63)**	P-Value*
Time of active labour (hours)	8.2	8.2	0.3
	(0.0-25.3)	(0.9-76.2)	
Time of pushing (minutes)	24	21	0.2
	(0.0-155.0)	(1.0-25.0)	
Labor dystocia (%)	337	27	0.6
	(46.5)	(42.9)	
Epidural analgesia (%)	369	37	0.2
	(51.0)	(58.7)	
Oxytocin (%)	386	35	0.7
	(53.4)	(55.6)	
***Mode of delivery***			
Vaginal (%)	525	54	0.03*
	(72.5)	(85.7)	
Operative (%)	199	9	
*Caesarean Section*	(27.5)	(14.3)	
**Reason for operative delivery**			
*Fetal distress (%)*	56	1	
	(28.1)	(11.0)	
*Dystocic labor (%)*	143	8	
	(71.9)	(89.0)	

Values are numbers (%) or medians (range).

* P-values <0.05 were considered as statistically significant.

An abnormal CTG trace during the last 30 minutes before delivery was common (480/787 = 61%) in the group with a higher BMI. Among newborns delivered with adverse fetal outcome at birth, 25/26 had a pathogical CTG trace 30 minute before delivery (p < 0.001). As part of the investigation of suspected fetal hypoxia during labour, fetal scalp blood samples were performed in 122/787 (15.5%) deliveries during the last 30 minutes before delivery. The numbers of fetal scalp blood samples performed in the two groups due to abnormal CTG trace was significantly higher in the group with a maternal BMI <30 compared with the group with a maternal BMI ≥30 (16.2 vs. 7.9%, p = 0.03) even if the registration was considered as abnormal, according to the definition, in both groups.

Adverse fetal outcome at delivery was statistically more common in the group with a maternal BMI ≥30 compared to those with maternal BMI < 30 (9.5 vs. 2.8%, p < 0.001). 28/787 (3.6%) newborns were admitted to NICU but no significant difference in the frequency of transfer was seen among the two BMI groups (p = 0.06). Neonatal outcome in deliveries with a maternal BMI < 30 compared to deliveries with a maternal BMI > =30 are described in Table 4.

**Table 4:** Neonatal outcome in deliveries with a maternal BMI < 30 compared to deliveries with a maternal BMI > =30, (n = 787)

	**BMI < 30 (n = 724)**	**BMI > =30 (n = 63)**	P-Value*
Apgar <7, 5’	1	7	<0.001*
(%)	(0.1)	(11.0)	
pH < 7.10	20	6	<0.001*
(%)	(2.8)	(9.5)	
pH <7.05	3	6	<0.001*
(%)	(0.4)	9.5)	
Metabolic acidosis**	2	5	<0.001*
(%)	(0.3)	(7.9)	
Fetal scalp blood sampled within 30 minutes before delivery (%)	117	5	0.03*
	(16.2)	(7.9)	
Normal CTG last 30 minutes of delivery	290	20	0.2
(%)	(40.0)	(31.7)	
Admission to NICU	23	5	0.06
(%)	(3.2.)	(7.9)	
Hypoglycemia	22	3	0.3
(%)	(3.0)	(4.8)	

Values are numbers (%) or median (range).

* P-values <0.05 were considered as statistically significant.

** Metabolic acidosis: arterial pH in cord blood <7.05 and BE > 12.

The maternal BMI was measured when attending the antenatal clinic in all deliveries included in the study. Among the 26 deliveries resulting in adverse neonatal outcome at birth, 23% (6/26) belong to the group with a maternal BMI ≥ 30. In the logistic regression analysis, maternal BMI ≥ 30 showed the most significant association with risk of delivering a neonate with an adverse outcome at birth (OR: 3.6, 95% CI; 1.4- 9.5, Table 1). Adjustment for all potential risk factors did not change the magnitude of the association between a high maternal BMI when attending the antenatal clinic, and the adjusted odds of delivering a neonate with an adverse fetal outcome. In our multivariate model, the odds of delivering a neonate with adverse fetal outcome were still about 4 times greater (OR: 3.6; 95% CI 1.4-9.8) if the maternal BMI was ≥ 30 when attending the antenatal clinic. Other risk factors such as maternal age, parity or smoking did not indicate any significant association with the measured outcome at delivery.

## Discussion

Obese women are considered as ‘high-risk’ obstetrical patients. Studies have shown that women suffering from obesity have an increased risk of a dystocic labour resulting in an operative delivery and an increased risk of obstetrical complications. Neonates delivered by an obese mother will more often be transferred to NICU care [2-8].

In this study we have shown that obese women (BMI ≥ 30), who are otherwise healthy and with normal pregnancies, more commonly deliver vaginally than women with a more normal body weight (BMI < 30). The result is slightly surprising as earlier publications have shown an overrepresentation of operative intervention among obese women. In this study, obese women had a higher proportion of abnormal CTG traces 30 minutes before delivery, but no significant statistical difference was shown. Only five deliveries with an abnormal CTG trace 30 minutes before delivery had fetal scalp blood sampled for analysis of the levels of lactate in fetal scalp blood, as expected by local guidelines. While a lower frequency of operative deliveries and fetal scalp blood sampling are noted among the obese women, a significantly higher frequency of newborns delivered with adverse fetal outcome was shown. Our data indicate that obstetrical problems sometimes appear to be ignored when handling the delivery of an obese woman. Although an abnormal CTG trace was present, only a few samples for analysis of lactate in fetal scalp blood were actually performed during the last 30 minutes before delivery, and most of the deliveries were terminated vaginally without interventions.

An increased frequency of operative deliveries due to labor dystocia has been reported among obese women [16]. Among the healthy but obese women in this study no such difference was indicated. The question is whether healthy women with obesity really have a higher incidence of dystocia, or whether it might be that the increased amount of intra-abdominal adipose tissue causes a slower labour progress? Should a slightly longer time of delivery be allowed among obese women without defining it as a dystocic labour? These theories are based on repeated measurements of scan fold thickness during pregnancy, and the findings are that more maternal fat is accumulated centrally than peripherally among obese women [23]. Our forthcoming studies will hopefully be able to answer the question of whether obese women will have a physiological explanation for the increased frequency of labour dystocia, described previously, or whether the major cause is the difference in obstetric management.

Some limitations of this study have to be mentioned. Fist to be discussed is the study design. In this project we looked at healthy women with or without obesity. This is a different way of analysing the problem compared to many other publications. Earlier publications are focusing on obese but nonhealthy women. The background is that we have in our clinical practice noted that we are acting a little bit different if the labouring women we are responsible for also have obesity. Secondly we found a lower incidence of obesity among the women included in this study than the incidence described in the national data in the Swedish Medical Birth Register [1,3]. A possible explanation may be that only a few women with high BMI will be totally healthy and will have a spontaneous onset of labour. Many obese women have pregnancy complications and have their deliveries induced or undergo a planned cesarean section. In this study, women with pregnancy complications have been excluded as they did not fulfil the inclusion criteria for the study. Only healthy women with a normal pregnancy and a spontaneous onset of labour remain. This selection bias may be the reason why the results we obtained differ from previous publications. Our purpose was in any event to avoid interference from complicating risk factors.

## Conclusion

In this study, obese but otherwise healthy women had a higher proportion of spontaneous vaginal delivery than women with a normal weight. However, these women also had an increased frequency of abnormal CTG registrations 30 minutes before delivery, fewer fetal scalp blood samples performed, and more newborns were delivered with adverse fetal outcome. These results may indicate that healthy but obese labouring women are at an increased risk at delivery and that the delivery management may not always be optimal. The question that arises is whether we as obstetricians/midwives are affected by the woman’s weight when we are handling her delivery. Consequently, these results should be replicated in future studies in other settings.

## Availability of supporting data

Supporting data has been deposited at Karolinska Institute, South Hospital and can be shown on request.

## Competing interests

Each author represents and warrants that she has no financial affiliation (eg, employment, direct payments, stock holdings, retainers, consultantships, patent-licensing arrangements, or honoraria) or involvement within the last 3 years with any commercial organization with a potential financial interest in the subject or materials discussed in the manuscript.

## Authors’ contributions

E-W-I initiated the study and collected the data. E-W-I, R KS and HÅ performed the data analyses. E-W-I, RK-S and HÅ drafted the manuscript and E-W-I, RK-S, HÅ, HV and LH-W contributed all to the revision of the manuscript and approved the final version.
